# Three Discipline Collaborative Radiation Therapy (3DCRT) Special Debate: I would treat prostate cancer with proton therapy

**DOI:** 10.1002/acm2.12621

**Published:** 2019-06-05

**Authors:** France Carrier, Yixiang Liao, Nancy Mendenhall, Patrizia Guerrieri, Dorin Todor, Anis Ahmad, Michael Dominello, Michael C. Joiner, Jay Burmeister

**Affiliations:** ^1^ Department of Radiation Oncology University of Maryland Baltimore MD USA; ^2^ Department of Radiation Oncology Rush University Medical Center Chicago IL USA; ^3^ University of Florida Proton Therapy Institute Jacksonville FL USA; ^4^ Department of Radiation Oncology Allegheny Health Network Pittsburgh PA USA; ^5^ Department of Radiation Oncology Virginia Commonwealth University Richmond VA USA; ^6^ Department of Radiation Oncology University of Miami, Sylvester Comprehensive Cancer Center, Miller School of Medicine Miami FL USA; ^7^ Department of Oncology Wayne State University School of Medicine Detroit MI USA; ^8^ Gershenson Radiation Oncology Center Barbara Ann Karmanos Cancer Institute Detroit MI USA

**Keywords:** prostate radiotherapy, protons, proton therapy

## 1 THREE DISCIPLINE COLLABORATIVE RADIATION THERAPY (3DCRT) DEBATE SERIES

Radiation Oncology is a highly multidisciplinary medical specialty, drawing significantly from three scientific disciplines — medicine, physics, and biology. As a result, discussion of controversies or changes in practice within radiation oncology involves input from all three disciplines. For this reason, significant effort has been expended recently to foster collaborative, multidisciplinary research in radiation oncology, with substantial demonstrated benefit.^1^, ^2^ In light of these results, we endeavor here to adopt this “team‐science” approach to the traditional debates featured in this journal. This article represents the fourth in a series of special debates entitled “Three Discipline Collaborative Radiation Therapy (3DCRT)” in which each debate team will include a radiation oncologist, medical physicist, and radiobiologist. We hope that this format will not only be engaging for the readership but will also foster further collaboration in the science and clinical practice of radiation oncology.

## 2 INTRODUCTION

Proton therapy has the ability to deliver exceptionally conformal dose distributions. This precision can be a double‐edged sword, providing the potential for remarkable sparing of adjacent normal tissues, but also the possibility of dramatic deviations from the intended dose distribution. The potential benefit of this improvement in dose distribution is dependent upon a variety of factors, many of which are specific to the individual treatment site. Uncertainties in these factors, particularly in prediction of the relative biological effectiveness, represent a major consideration in the applicability of proton therapy. The treatment of prostate cancer represents a significant fraction of all radiotherapy treatments, however, the tangible benefits of the use of proton therapy for prostate cancer are still hotly debated. This is the subject of this month's 3DCRT debate.

Arguing for the proposition will be Drs. France Carrier, Yixiang Liao, and Nancy Mendenhall. France Carrier, PhD, is a Professor of Radiation Oncology within the School of Medicine at the University of Maryland. Dr. Carrier has published more than 50 peer reviewed scientific articles that have been cited over 7,000 times. Her research interests include the rational design of small molecule inhibitors of protein translation (NCI 1R01CA177981‐01) and chemopotentiation by Low‐Dose Fractionated Radiation Therapy (VA merit award).

Yixiang Liao, PhD, is an Assistant Professor of Radiation Oncology at Rush University Medical Center in Chicago and serves as the associate director of the medical physics residency program. Dr. Liao has published in Red Journal on the hypofractionation in prostate cancer treatment. She is among the first‐year students of the IBPRO (Integrated course in Biology and Physics of Radiation Oncology).

Nancy Mendenhall, MD, Medical Director, UF Health Proton Therapy Institute has been a University of Florida College of Medicine faculty member since 1985, serving as the Department of Radiation Oncology chair 1993‐2006. She is a leader in research, has extensive experience in cooperative group trials (COG) and has produced more than 275 published works, including articles in such publications as the Journal of the American Medical Association, International Journal of Radiation Oncology, Biology, and Physics, Cancer, Acta Oncologica, American Journal of Clinical Oncology, and the International Journal of Particle Therapy.

Arguing against the proposition will be Drs. Patrizia Guerrieri, Dorin Todor, and Anis Ahmad. Patrizia Guerrieri, MD, is board certified in Radiation Oncology in Italy and the USA and has a MS in Radiation Sciences. She currently practices at Allegheny Health Network in Pittsburgh and has particular expertize in HDR brachytherapy, IMRT, and SBRT, for Head/Neck, Breast, and Gynecological cancers. She has authored publications, abstracts, and book chapters on gynecological brachytherapy, altered fractionation, and brachytherapy in the elderly and was a contributor to the Radiation Oncology Encyclopedia as well as “Principles and Practice of Radiation Oncology” by Perez and Brady.

Dorin Todor received his PhD from Old Dominion University, followed by a postdoctoral fellowship at MSKCC. He is now an Associate Professor with Virginia Commonwealth University Health System and Director of Brachytherapy Physics service. He is currently the chair of the ABS physics committee and serves as Associate Editor for Medical Physics and Brachytherapy journals. Dr. Todor's main research interests are biological effect modeling, optimization, and brachytherapy.

Anis Ahmad, PhD, received his MPhil and PhD from Aligarh Muslim University, India followed by a postdoctoral fellowship at the Medical University of South Carolina. He has authored more than 30 peer‐reviewed scientific articles, and has been cited over 900 times. He serves as Associate Editor for the Open Access Journal of Cancer & Oncology and review editor for Frontiers in Neurodegeneration. He is now an Assistant Scientist with Sylvester Comprehensive Cancer Center at the University of Miami. Dr. Ahmad's primary research focuses are radiation response of tumor and normal tissue to low and clinically relevant doses of radiation.

## 3 OPENING STATEMENTS

### 3.1 France Carrier, PhD; Yixiang Liao, PhD; Nancy Mendenhall, MD

#### 3.1.1 Physics

The fundamental advantage of protons as a modality for radiation therapy in prostate cancer is related to the physical properties of a proton beam resulting in improved radiation dose distribution. A photon beam deposits dose from skin entrance to skin exit, leaving a track of damage much like a bullet. After the depth of maximum dose deposition, which occurs within a few cm below the skin surface, the dose is attenuated with increasing depth as the photon beam exits the patient. Thus entrance dose with a photon beam is generally substantially more than target dose and beyond the target there is dose deposition until the beam exits the patient. Most of the radiation dose is actually deposited outside the target, rather than inside the target, with photon‐based radiation therapy. Unlike photons, protons are particles with mass and travel only to a finite depth in tissue, proportional to their acceleration; most of the energy deposited by a proton beam occurs just before the end of the proton range in a pattern known as the Bragg peak. Beyond the Bragg peak, there is essentially no dose deposition; before the Bragg peak, along the entrance path, there is a constant relatively small dose deposition compared with the Bragg peak. The end of range can be controlled through varying the acceleration of the protons, thus the Bragg peak can always be placed in the target, regardless of the target depth. With no exit dose and much less entrance dose compared to target dose, most of the radiation dose deposited with a proton beam is in the target rather than in nontargeted tissues, as with photon‐based therapy, resulting in significantly reduced integral dose. Less integral dose should lead to less early and late toxicity (including rectal and bladder damage and second malignancies).^3^ Less integral dose should also facilitate dose escalation and/or intensification (hypofractionation) which should lead to enhanced disease control and/or reduced expense. In the case of prostate cancer, proton therapy usually deploys a simple plan consisting of two lateral beams and achieves the desired target coverage and OAR sparing as well as the conformity comparable to the much more complicated photon external beam plans.^4^, ^5^ From the physics perspective, the marked reduction in integral dose and simplicity of treatment plan make proton therapy the logical choice for prostate cancer. A potential concern regarding proton therapy from the physics perspective has been the ability of treatment planning systems to account for range and RBE uncertainties.^6^, ^7^

#### 3.1.2 Biology

Prostate tumors have the lowest α/β ratio of any human tumor because of unusually long cellular doubling times, from 15 to more than 70 days.^8^, ^9^ This means that with prostate cancers, there is a lot of repair between fractions, little repopulation, minimal redistribution, and minimal reoxygenation.^10^, ^11^, ^12^ Therefore, prostate cancers should demonstrate enhanced disease control with the large fractional doses used in hypofractionation regimens.

In addition, in vitro studies have shown increased “relative biologic effectiveness” for proton therapy compared with photon therapy, particular at the end of the range.^13^ The ionization and molecular excitation patterns are densely concentrated along the path of protons in contrast to sparsely distributed events across a field irradiated with photons. As a consequence, proton therapy produces greater complexity of DNA damage which requires different mechanisms for DNA repair. This may lead to enhanced disease control compared with photon‐based therapy.^11^, ^12^, ^13^, ^14^, ^15^, ^16^, ^17^ In addition, the gene expression responses suggest that protons may result in greater downregulation of certain genes that could impact metastases.^18^

Prostate cancer should benefit from hypofractionation and proton therapy, with less integral dose, and should provide a safer method of hypofractionation. There is also increasing evidence for differential molecular excitation patterns, DNA repair mechanisms, and signaling responses that may result in enhanced disease control and reduced distant metastases compared with photon therapy. Proton therapy is therefore logical choice for prostate cancer from the biology perspective.

#### 3.1.3 Clinical outcomes

Because of the paucity of operating proton facilities, there are only a few large published clinical experiences in prostate cancer. The outcomes with respect to toxicity and disease control in prostate cancer are remarkably similar between these large proton experiences: grade 3 GI and GU toxicity rates appear to be on the order of 0.5% and 1–3% and disease control (freedom from biochemical progression [FFBP]) rates for low and intermediate risk disease have been on the order of 99% and 95% at 5 yr.^4^, ^19^, ^20^, ^21^ Although there are variations between the series in the toxicity scoring systems used to report toxicity, FFBP is a relatively objective surrogate for disease control, suggesting that the clinical significance of range and RBE uncertainties with proton therapy has been overestimated.^6^, ^7^ In addition, the 5 yr FFBP rates with standard radiation fractionation contemporary photon therapy of 92‐98% for low risk and 85‐86% for intermediate risk^22^, ^23^ appear to possibly be slightly inferior to reported FFBP rates of 99% for low risk and 95% for intermediate risk with proton therapy, suggesting the possibility of enhanced disease control with proton therapy, although prospective controlled studies would be required to determine whether factors of patient selection, treatment technique, dose or dose per fraction variations, rather than biologic effectiveness accounted for these historical outcomes. Furthermore, outcomes from hypofractionated photon therapy in low‐risk prostate cancer^24^ include grade 3 GI and GU toxicity rates of 4% and 3.5% and 5 yr FFBP rates of 86% while contemporaneous outcomes from hypofractionated proton therapy in low‐risk prostate cancer include grade 3 GI and GU toxicity rates of < 1% and 2% and 5 yr FFBP rates of 99%.^25^ The identical dose fractionation schemes and the absence of adjuvant hormone therapy in these contemporaneous proton and photon hypofractionation series suggest the possibility that, as physics predicts, reduced integral dose will lead to safer hypofractionation with proton therapy and that, as biology predicts, protons may be more effective than photons, especially in hypofractionated regimens.

Although these early clinical observations are concordant with predictions based on the physics and biology of proton therapy, they must be tested prospectively in a controlled trial. In the absence of results from a well‐designed comprehensive controlled clinical trial simultaneously assessing toxicity, patient‐reported outcomes, and disease control, the current rationale based on the physics and biology of photon and proton interactions in tissue and the current clinical data make a compelling argument for proton therapy in prostate cancer.

### 3.2 3.B Patrizia Guerrieri, MD; Dorin Todor, PhD; Anis Ahmad, PhD

Treatment for clinically localized prostate cancer spans a large range of options, from active surveillance, multiple surgical approaches to prostatectomy, various forms of external beam and interstitial radiation, and a growing number of ablative methods, employing heat and cold. Within radiation therapy, treatment options include external beam radiation therapy (RT), which may be conventionally fractionated (CFRT) with intensity modulated radiation therapy (IMRT), protons or intensity modulated protons therapy, hypofractionated RT (HFRT) with IMRT or protons (IMPT), or delivered as stereotactic body RT (SBRT); and brachytherapy (BT), either high‐dose rate (HDR‐BT) or low‐dose rate (LDR‐BT).

The metrics used to compare these modalities are treatment efficacy, through cure rates and mortality, as well as complications, side effects, and financial costs.

The debates for or against protons are already numerous and have generated a long laundry list of reasons on why protons are NOT better than photons for most common sites like breast and prostate, from a clinical, physics, radiobiological, and economical perspective. While radiation is widely acknowledged as effective in the treatment of prostate cancer, there is no inherent biological basis to believe that 1 Gy of photon radiation would be any different from a comparable adjusted Gy delivered with protons. Based on cross‐institutional studies, evidence for benefits favoring proton beams do not exist. Similarly, there are no large quality of life studies concluding that protons produce a better quality of life profile than photons. It remains to be proved that protons are a more effective treatment. Without reiterating here the arguments already made in previous debates, we will recognize that the available dosimetry models fall short of converting a physical dose distribution into a clinical effect, that would take into account all the different biological components involved. Just only recently, in fact, we are starting to better understand and exploit the role of biological mechanisms that may be triggered by radiation and its role as an immunomodulator.^26^

The application of proton therapy to prostate cancer remains one of the most controversial issues within radiation oncology from many perspectives, starting with the radiobiological one.^27^, ^28^, ^29^, ^30^ The radiobiological studies tend to emphasize the issues regarding relative biological effectiveness (RBEs) for protons as a critical point. While typical photons‐protons comparisons include metrics like normal tissue complication probabilities (NTCP) and the expected tumor control probability (TCP), one should point out that none of the current models have provisions for handling spatial dose inhomogeneity at micro‐ or macro‐scale, nor do they take into account any other effect than “cell kill.”.

As the dose deposition at the microscopic scale is fundamentally different between photon and proton radiotherapy, the biological equivalent dose is usually compared by using a constant RBE of 1.1 for protons,^31^ thus providing a slightly different or “adjusted” total dose. However, the nature of RBE makes it dependent on both the dose and the chosen tissue and endpoint (through α/β), as well as on parameters such as the linear energy transfer (LET).^32^ Taking the variable RBE into account may generate heterogeneous RBE distributions that could degrade the advantageous proton dose distribution, as shown in the study by Wedenberg and Toma‐Dasu.^33^ For low α/β, such as local control for prostate,^34^ it is of special concern to account for the variable RBE as the 1.1 constant factor is likely to underestimate the biological effect, especially for low doses.^32^, ^35^

Relative biological effectiveness‐based IMPT approaches need to be taken with caution. It has been shown that such optimization could lead to sub‐optimal plans due to RBE uncertainties.^32^ If the RBE is overestimated, the target could be significantly underdosed, while an underestimation of the RBE value in the OARs could lead to significant organ toxicity. Until in vivo verification of RBE models is available, such implementations of RBE‐based IMPT planning might be premature. Most proton RBE models are derived from the linear‐quadratic dose‐response model and use α/β to characterize tissue radiosensitivity^36^ predicting a higher RBE for a low α/β tissue like prostate cancer.

Quantifying the dependencies of RBE, LET and α/β is challenging due to differences in patient radiosensitivity. These include genomic factors and tumor heterogeneity in DNA repair pathways that influence the RBE,^37^ or the different presence of growth or modulatory receptors. In consequence, the currently used constant factor of 1.1 might lead to an underestimation of the real biological equivalent doses, especially for conventional fractionation schedules of around 2 Gy (RBE) per fraction.

From a physical point of view photons, protons, and heavy particles elicit different mechanisms of actions starting at the atomic level and give rise to different spatial dose distribution patterns.^38^

In the case of the prostate, the Bragg peak might represent an advantage in unidirectional sparing predefined OARs, but the central location of the prostate and the lateral incidence of the proton beams implies for the beams the need to be degraded to reach a homogeneous dose distribution while failing in their conformality when compared to photons.^39^ Goddard showed that even when using hypofractionation to treat prostate cancer, VMAT is still superior to IMPT in terms of target conformity and OARs sparing,^40^ while interfractions and intrafractions organs motion still represent a problem in proton delivery.^41^ It is interesting that in their zeal of finding usefulness for protons, investigators now point out that while the high‐dose areas in OARs are not any better with protons, maybe the low‐dose areas (where protons presumably offer some advantage) are the ones instrumental to injury!.^42^

In the clinic, the only randomized trial comparing protons and photons is the one from 1995 from Shipley that is, in reality, a study of dose escalation.^43^ Other more recent trials have compared protons to radical surgery finding no differences in local control, while there are, so far, no strong clinical data verifying the claimed advantage of protons over photons in terms of side effects. In terms of toxicity, in fact, some authors believe that protons cause less side effect, while others argue the contrary; in reality, by looking at grade 2 or 3 + late side effects, the results are very similar, for both GI and GU toxicity, among pencil‐beam protons (RBE about 78 Gy) and hypofractionated IMRT (60 Gy in 20 fractions), which are two of the most used schedules in clinical practice.

Finally, when we evaluate the cost‐effectiveness of protons over photons, especially of IMPT versus IMRT, there is no doubt that the cost according to QALY parameters favors the use of IMRT for prostate cancer.^44^ This, together with studies showing that a more extensive use of brachytherapy, not only in low risk, but also in higher risk prostate cancer, as a way to dose escalate, while optimally sparing the OARs, has shown important benefits in terms of loco‐regional control and quality of life, set the parameters against which proton therapy needs to be compared.

In summary, we conclude that because of the not clear dosimetric advantage, the complex and not completely understood radiobiological issues, the lack of a real sparing of side effects and the cost of using protons, protons do not represent a treatment of choice in prostate cancer, compared to the wide range of other available alternatives.

## 4 REBUTTAL

### 4.1 France Carrier, PhD; Yixiang Liao, PhD; Nancy Mendenhall, MD

#### 4.1.1 Physics

Our Con‐proton opponents argue that the conformity of lateral proton beams will be degraded to be worse than photon beams for a deeply seated prostate cancer. Kooy et al.^39^ did point out that the pencil beam brush size for lateral beams in prostate cancer treatment is on the order of 10mm. However, Kooy et al also point out that a smaller effective spot can be easily achieved by edge collimation with an aperture and in their thorough review of the advances in IMPT, they concluded that benefit of proton therapy is reduction in the integral dose bath which affects tissues outside the planning target volume (PTV) in all disease sites, especially for bigger target volumes.

The opponents' argument that target conformity and OAR sparing in IMPT are inferior to VMAT is questionable because the referenced article used a nonconventional proton beam configuration (two anterior‐oblique and one posterior) not commonly used in proton clinics, as pointed out by Paganetti et al.^42^

Inter‐ and intra‐fractional organ motion poses similar challenges to both proton therapy and IMRT: Moteabbed et al.^41^ found no statistically significant differences in DVH indices between passive‐scattering proton therapy and IMRT.

Our opponents also argue that the higher RBE in the low‐dose region could cause harm to normal tissue. The reference they cite actually points out that higher RBE is predicted in prostate cancer because of the lower α/β of prostate cancer compared to its surrounding OARs,^45^ which makes it the ideal candidate for proton therapy due to the expected inverse correlation of RBE and α/β, which would predict an increased RBE in tumor and a reduced risk of side effects in OARs.

#### 4.1.2 Biology

The inherent biological differences between photon and proton therapy are based on the protons' pattern of energy deposition as stated in our opening statement. This results in increasingly clustered DNA damage that is more difficult to repair and consequently enhanced cellular death as well as different gene expression that could impact metastases. We agree that the generic use of proton RBE of 1.1 in all tissue types at all doses and LET values is not an optimal ratio for proton dose modification. However, the debate regarding this ratio is still ongoing for many reasons including the fact that it was derived almost entirely from clonogenic cell survival assays of early reacting tissues.^46^ In prostate cancer, the unusually low α/β ratio (1.5 Gy) is more reminiscent of late reacting tissues than most tumors types (~10 Gy) and is still lower than the α/β ratio of late‐responding normal rectal tissues (~3 Gy). A recent study performed on six prostate cancer patients actually demonstrated that the low α/β ratio of the prostate translated into a higher biological dose in the target than predicted with a RBE of 1.1.^47^ On the other hand, three variable RBE models predicted higher estimates of rectum and bladder normal tissue complication probabilities (NTCP).^48^ Our understanding of the proton radiobiological effect is still limited. As the number of patients treated with PT increases, it will be imperative to recalculate NTCPs based on actual PT experiences and perform well controlled experiments to better describe and model biologic effects of proton beams.

#### 4.1.3 Clinical

As discussed above, the Con‐Proton Debaters have concluded there is a “not clear dosimetric advantage” for PT in prostate cancer compared with IMRT. We agree there may be no advantage with PT for normal tissues, such as the anterior rectal wall, that are included in the PTV; however, the dosimetric advantage of PT for normal tissues not included in the PTV (such as the entire rectal volume, the bladder volume, the penile bulb, the pelvic tissues at risk for second malignancy) are obvious. The correlation between side effects in these tissues and the dosimetric differences between PT and IMRT are poorly defined at this point, but modeling data suggests there will indeed be fewer second malignancies with PT^3^ and clinical data suggests a reduction in second malignancy by at least 50%.^49^ Testosterone suppression has been shown to be less with PT^50^ and quality of life clinical outcome data suggests significant differences in bowel urgency and bowel frequency which dosimetric differences would predict.^51^

As discussed above, there are indeed unknowns in the biology of PT, but this is not new in radiation oncology; the proof has always been in the pudding. Outcomes of very large historical series with both standard and moderate hypofraction in prostate cancer^4^, ^19^, ^20^, ^21^, ^25^ appear somewhat better than outcomes with contemporary photon‐based therapy.^22^, ^23^, ^24^ To be certain, a head‐to‐head comparative trial is necessary; two are underway.^52^, ^53^ As we await these trials, we must be careful about considering “cost‐effectiveness” as a primary comparator. It is difficult to calculate “cost‐effectiveness” when there is no intervention (and thus no cost) for a treatment decision that results in major quality of life issues such as bowel urgency or frequency. Without > 10‐yr follow‐up with either IMRT or PT, it is difficult to confirm dosimetric predictions regarding second malignancies. When early outcomes with PT appear better, waiting to offer PT for documentation of additional improved late effects because of higher early costs seems questionable. There is no reason not to choose proton therapy in prostate cancer if one has access to it.

### 4.2 Patrizia Guerrieri, MD; Dorin Todor, PhD; Anis Ahmad, PhD

At first analysis, protons do indeed appear to have a dosimetric theoretical advantage. At further scrutiny though, a litany of factors dilute any such potential theoretical advantage. Among these factors, uncertainty due to the conversion of electron densities, measured using a CT scan, to proton stopping powers has the potential to completely miss a distal part of the tumor or alternatively, delivering high dose to adjacent OAR. The sensitivity of path length to tissue heterogeneity is particularly of concern for a deep mobile target like prostate where variations in bladder and rectal filling can be significant.^30^ Typical beam arrangements leading to high scatter and wide penumbra, the intra‐ and interfraction motion and setup errors all lead to the use of larger margins and decreased conformity, making “improved dose distribution” a somehow distant goal.

The advantage in terms of dose deposition needs to compete with the already excellent results of IMRT in terms of tumor outcome and side effects. The majority of the data come from noncomparative cohort studies and few retrospective comparative studies of patient‐reported QOL/toxicities and they do not really show a superiority of protons compared to photons in prostate cancer, for the biological and physics problems connected with this technique in prostate cancer, and for the extreme competition in terms of dose escalation offered by brachytherapy that is proving its efficacy not only in intermediate/low risk but also in high‐risk patients.^54^, ^55^

The argument that “prostate cancers should demonstrate enhanced disease control with the large fractional doses used in hypofractionation regimens” while correct, it points out again to brachytherapy, where super high doses are routinely delivered in the most conformal manner, with greater accuracy and without motion or setup uncertainties.

While typically the larger than unity RBE is used as a “pro” argument, the reality is, well, complicated. Early studies^56^ showed that the average RBE value at mid‐SOBP in vivo is approximately 1.1, the generic value typically used, but ranging from 0.7 to 1.6, with the hot region over the terminal few millimeters of SOBP (Spread Out Bragg Peak). Later studies^57^ concluded that “the RBE of a high‐energy proton beam and the cellular responses, including the DNA damage repair processes, to high‐energy proton beam irradiation, differ according to the position on the SOBP, irrespective of the radiosensitivity levels of the cell lines” showing that including a variable RBE in a treatment plan is difficult, and validating it in vivo is absolutely necessary for any real comparison with IMRT. A recent study^58^ on the effect of variable RBE models on spot scanning treatment plans predicted increased biological doses to rectum, bladder, and prostate leading to higher NTCP estimates for bladder and rectum.

The hypothesis that somehow, a greater complexity DNA damage induced by protons may lead to enhanced disease control and positively impact the probability of metastases is not new. Numerous publications have investigated photon radiation‐altered migration and invasion; however, data on the effect of particle radiation are still limited.^59^ Further work is needed to implement proton therapy in combination with anti‐angiogenic or anti‐immune checkpoint drugs. It is not clear whether the theoretical benefits of proton beam therapy could be translated into clinically meaningful improvement for prostate cancer patients, so any progress implies an urgent need for prospective randomized clinical trials to measure the toxicity and disease control.^60^

In conclusion, in place of a discussion that interests only the radiation oncology community, we think that we need to look at a bigger picture. We believe that protons have proven advantageous in many clinical situations and will prove their efficacy in many tumor sites, but prostate is probably not the best paradigm for their use, due to other available great alternatives. Therefore we would not recommend installing proton facilities based almost exclusively on prostatic cancer numbers. If there is an existing facility for protons, we believe it would be right to treat prostate with protons as well, especially by implementing geometric configurations different from the classical latero‐lateral beams, that might potentially allow for a better dose conformality; but only in the optic of widening the indication to protons in that particular center. We need to recognize the increased cost of protons and discuss with insurance companies novel models of reimbursement where the need for more sophisticated techniques meet midway with the need to contain the healthcare costs. We also need to abide by our own expertize as radiation oncologist and promote and not lose the capabilities of using procedures that are at the core of our profession, like brachytherapy, along with the advances in technology. We need to divert our gaze from our computers to be able to look at the complexity of our patients' care and be able to put our specialty center stage in the battle against cancer, one more time.

Even more important, these types of debates between technical and technological modalities should not precede real debates and questions central to actual progress in our field: how can we better understand and model a more realistic dose effect, including spatial dose distribution, structure and function of irradiated tissues, role of dose inhomogeneity and irradiation time, role of the immune system, etc. While some progress has been made in each of these separate topics, it is unlikely that real progress can be made continuing to create plans based on DVH parameters.

## CONFLICTS OF INTEREST

The authors declare no conflicts of interest.
